# A Conserved Class II Type Thioester Domain-Containing Adhesin Is Required for Efficient Conjugation in Bacillus subtilis

**DOI:** 10.1128/mBio.00104-21

**Published:** 2021-03-16

**Authors:** César Gago-Córdoba, Jorge Val-Calvo, David Abia, Alberto Díaz-Talavera, Andrés Miguel-Arribas, Rocío Aguilar Suárez, Jan Maarten van Dijl, Ling Juan Wu, Wilfried J. J. Meijer

**Affiliations:** a Centro de Biología Molecular Severo Ochoa (CSIC-UAM), Universidad Autónoma, Madrid, Spain; b University of Groningen, University Medical Center Groningen, Groningen, The Netherlands; c Centre for Bacterial Cell Biology, Biosciences Institute, Newcastle University, Newcastle upon Tyne, United Kingdom; GSK Vaccines

**Keywords:** adhesion molecules, antibiotic resistance, conjugation, Gram-positive bacteria, mating, plasmids, thioester domain

## Abstract

Conjugation, the process by which a DNA element is transferred from a donor to a recipient cell, is the main horizontal gene transfer route responsible for the spread of antibiotic resistance and virulence genes. Contact between a donor and a recipient cell is a prerequisite for conjugation, because conjugative DNA is transferred into the recipient via a channel connecting the two cells. Conjugative elements encode proteins dedicated to facilitating the recognition and attachment to recipient cells, also known as mating pair formation. A subgroup of the conjugative elements is able to mediate efficient conjugation during planktonic growth, and mechanisms facilitating mating pair formation will be particularly important in these cases. Conjugative elements of Gram-negative bacteria encode conjugative pili, also known as sex pili, some of which are retractile. Far less is known about mechanisms that promote mating pair formation in Gram-positive bacteria. The conjugative plasmid pLS20 of the Gram-positive bacterium Bacillus subtilis allows efficient conjugation in liquid medium. Here, we report the identification of an adhesin gene in the pLS20 conjugation operon. The N-terminal region of the adhesin contains a class II type thioester domain (TED) that is essential for efficient conjugation, particularly in liquid medium. We show that TED-containing adhesins are widely conserved in Gram-positive bacteria, including pathogens where they often play crucial roles in pathogenesis. Our study is the first to demonstrate the involvement of a class II type TED-containing adhesin in conjugation.

## Abstract (precis)

Bacterial resistance to antibiotics has become a serious health care problem. The spread of antibiotic resistance genes between bacteria of the same or different species is often mediated by a process named conjugation, where a donor cell transfers DNA to a recipient cell through a connecting channel.

## INTRODUCTION

Bacteria exchange DNA at large scale by different routes which are collectively called horizontal gene transfer (HGT) (for review, see references [Bibr B1][Bibr B2][Bibr B4]). HGT shapes the genetic content and plays a major role in the evolution of bacteria. The downside of HGT is that it contributes importantly to the emergence and dissemination of antibiotic resistance (5), which is one of today’s major health care problems (6).

Conjugation, the process by which a conjugative DNA element is transferred from a donor to a recipient cell via a connecting channel, is the principal HGT route that is responsible for the spread of antibiotic resistance (5, 7, 8). Conjugative elements that are inserted in a bacterial genome are called integrative and conjugative elements (ICEs), and those present on plasmids are named conjugative plasmids. The basic principles of conjugation are conserved among ICEs and conjugative plasmids present in Gram-negative (G^−^) or Gram-positive (G^+^) bacteria. Conjugation occurs when cells are in close contact with each other, for instance in biofilms. However, a subset of conjugative elements is able to mediate conjugation with high efficiency also during planktonic growth. A prerequisite for conjugation is that donor cells recognize and contact a recipient cell in a process named mating pair formation (MPF). Particularly during conjugation in liquid medium, the presence of adhesive molecules or organelles is important for establishing contact between donor and recipient cells. Sophisticated type IV secretion systems (T4SS) are known to connect the donor and recipient cells, through which a copy of the conjugative DNA element is transported. All conjugative T4SS of G^−^ bacteria involve pili, also known as sex pili, which extend from the donor surface into the extracellular space and are involved in recipient cell recognition. Among the best studied are the pili encoded by the Agrobacterium tumefaciens pTi and the Escherichia coli F plasmids, named T- and F-pilus, respectively. The biogenesis and function of the T-pilus have recently been reviewed (9, 10). The T-pilus is formed as an extension at the surface of the T4SS and consists of multiple VirB2 shaft subunits and a VirB5 pilus tip adhesin protein. F-pili are long, flexible, and occasionally retractile. Their synthesis is more complex than that of T-pili (for review, see references [Bibr B11][Bibr B12][Bibr B13]).

Much less is known about how G^+^ donor cells attach to recipient cells, but it seems that T4SS of G^+^ bacteria do not develop pili. The only T4SS of a G^+^ system for which recipient attachment has been studied in detail is PrgB encoded by the Enterococcus faecalis conjugative plasmid pCF10 (for review, see reference 14). Recently, the structure of the PrgB adhesin domain was shown to resemble the lectin-like fold in general and the multimodal *Streptococcus* sp. AglI/II, SspB, and SpaP adhesins in particular (15).

Conjugative plasmid pLS20 from the G^+^ bacterium Bacillus subtilis and its derivative pLS20cat, which contains a chloramphenicol resistance gene, conjugate efficiently in both solid and liquid media (16–18). We therefore reasoned that pLS20 would encode a protein(s) that allows efficient MPF, especially in liquid media. pLS20 contains a large conjugation operon encompassing genes *28* to *74* (according to our gene annotation [19]). Expression of the conjugation operon is controlled by a strong promoter, P_c_, whose activity is regulated by proteins encoded by genes *25* to *27* (19, 20). Here, we show that in the absence of pLS20cat gene *34* conjugation was severely affected in liquid media and moderately in solid media. Gene *34* encodes a 778-residue-long protein with a predicted N-terminal signal peptide. We present evidence that protein p34 is an adhesin that contains a class II type thioester domain (TED). A point mutation predicted to prevent formation of the thioester bond rendered the protein inactive. Sequence similarity and modeling strongly indicate that the TED is followed by CnaB-type structures with intramolecular isopeptide bonds that function potentially as a stalk to position the TED-type adhesin away from the cell surface. Therefore, protein p34 belongs to a family of G^+^ bacterial proteins that are characterized by intramolecular cross-links in structurally conserved thioester, isopeptide, and ester domains and are hence named TIE proteins. Correspondingly, we have named p34 TIE_pLS20_. TIE proteins have previously been shown to play important roles in the virulence of various pathogenic bacteria. Our results show, for the first time, that a TED-containing adhesin also plays an important role in plasmid conjugation.

## RESULTS

### pLS20cat gene *34* is required for efficient conjugation in liquid medium.

**FIG 1:**
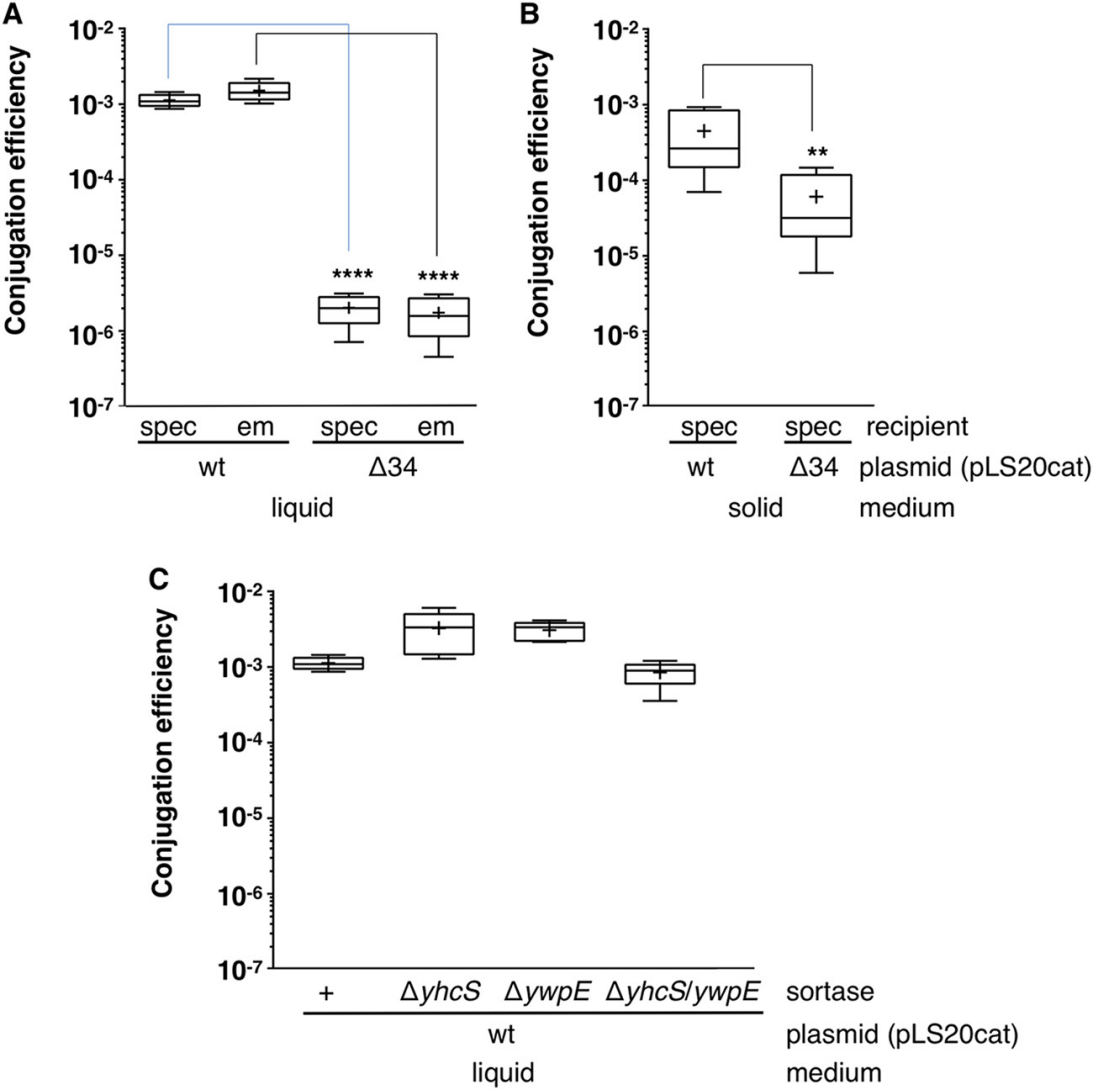
The absence of pLS20cat gene *34*, but not the sortase genes *yhcS* and *ywpE*, affects conjugation efficiency more severely in liquid than in solid medium. (A and B) Conjugation efficiencies were determined for the wild-type (wt) plasmid pLS20cat (strain PKS11) and pLS20catΔ34 (strain CG164) in liquid (A) or solid (B) medium. To test possible differences due to the presence of different antibiotic markers, two strains with different resistance genes, PS110 (spectinomycin [spec] resistant) and PKS7 (erythromycin [em] resistant), were used as recipient strains in the liquid medium experiments. (C) Conjugation efficiencies in wild-type and sortase-deficient *yhcS* and/or *ywpE* donor strains. Conjugation efficiencies were calculated as the number of transconjugants per donor cell. Each experiment was repeated at least five times. Data are shown as box plot graphs. The box is determined by the 25th and 75th percentiles, and whiskers are determined by 5th and 95th percentiles; the line in the box indicates the median, and the “+” symbol indicates the mean for each sample data set. Analyses of variance (ANOVAs) show that the obtained differences in conjugation efficiencies between pLS20cat and pLS20catΔ34 are statistically significant with *P* values of *P* < 0.0001 (****) and *P* < 0.01 (**) for liquid and solid media, respectively. No significant differences were observed between different recipient strains used in liquid medium or in the sortase-negative strains (*P* > 0.05).

In our recent study on exclusion proteins, we screened all pLS20cat genes for candidates encoding putative surface proteins (21). Besides the exclusion gene *ses_pLS20_* (gene *29*), gene *34* was also predicted to encode a surface protein. In the two plasmid sequences deposited in the NCBI database (accession numbers AB615352.1 and NC_015148.1), pLS20cat gene *34* is annotated as a gene encoding a 753-residue protein (BAJ76911.1 and YP_004243498.1, respectively). However, the proposed GTG start codon is not preceded by a good ribosomal binding site. Importantly, the reading frame could be extended at the 5′ end for 25 codons, where another putative GTG start codon is preceded by a ribosome binding site (RBS) (AAAGGGG-8 bp-GTG). The deduced sequence of the longer version of protein p34 (778 residues) contains an N-terminal signal peptide, indicating that the protein is exported from the cytoplasm, which is consistent with p34 sharing (low-level) similarity to a number of adhesins (see below). Based on this, we hypothesized that p34 could be an adhesin with a role in MPF. As a first strategy to test this idea, we constructed a derivative of pLS20cat, named pLS20catΔ34, containing a large in-frame deletion in gene *34*. Strain CG164 harboring pLS20catΔ34 was employed as a donor in conjugation experiments using standard conditions to determine conjugation efficiency in liquid medium. In parallel, conjugation experiments were performed using as donor strain PKS11 harboring wild-type pLS20cat. In agreement with previous results, conjugation efficiencies in the range of 1 × 10^−3^ (calculated as transconjugants per donor) were obtained for pLS20cat (Fig. 1A). However, about ~1,000-fold-lower conjugation efficiencies were obtained for pLS20catΔ34, demonstrating that gene *34* is required for efficient conjugation in liquid medium. In these experiments, the spectinomycin-resistant strain PS110 was used as recipient. To verify that the antibiotic resistance marker had no effect on conjugation efficiencies, we repeated these experiments using as recipient the erythromycin-resistant strain PKS7. Similarly reduced conjugation efficiencies were obtained when gene *34* was disrupted regardless of the antibiotic marker used (Fig. 1A).

Next, we investigated whether gene *34* was also required for efficient conjugation on solid medium. Importantly, although inactivation of gene *34* also resulted in lower conjugation efficiencies on solid medium, the effect was much smaller than that observed in liquid medium (Fig. 1B). Compared to pLS20cat, the conjugation efficiencies of pLS20catΔ34 were about 1,000- and 15-fold lower in liquid and solid media, respectively. Together, these results demonstrate that pLS20cat gene *34* is required for efficient conjugation, particularly in liquid medium.

### Ectopic expression of pLS20cat gene *34* in donor cells, but not in recipient cells, restores efficient conjugation of pLS20catΔ34.

**FIG 2:**
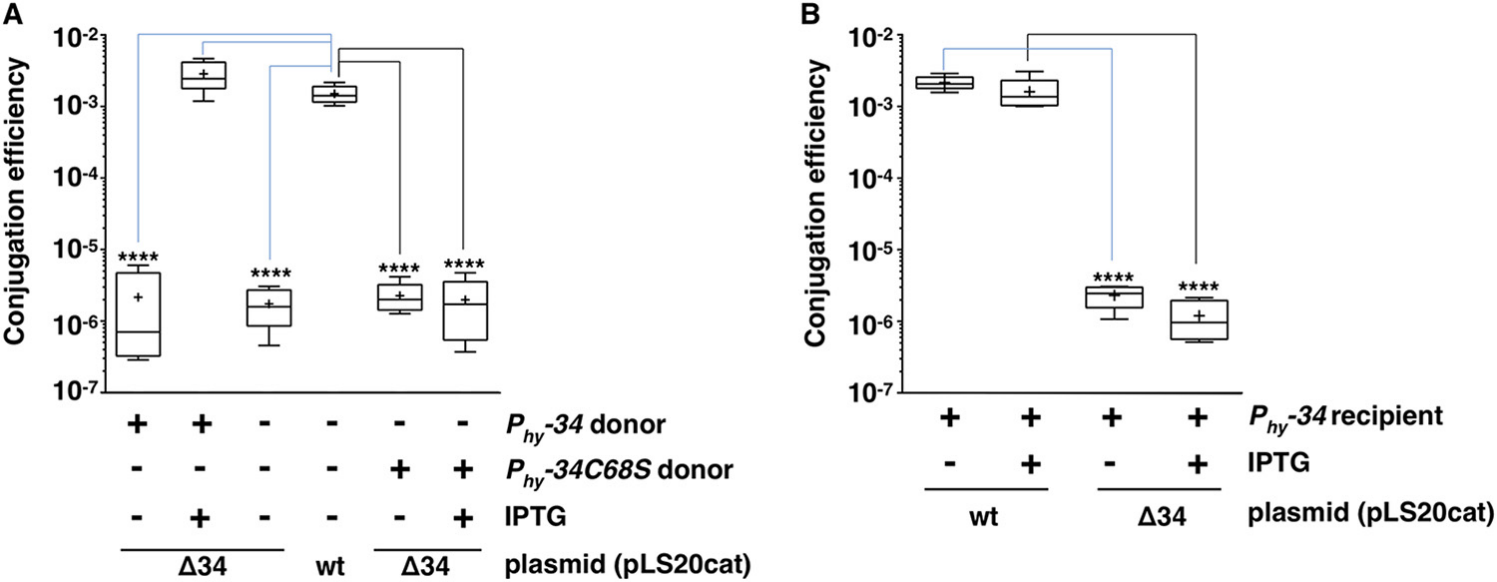
Effects of ectopic expression of the wild-type or the *34C68S* mutated version of gene *34* in donor cells or recipient cells on pLS20cat and pLS20catΔ34 conjugation. Conjugation efficiencies were determined in liquid medium for pLS20cat and pLS20catΔ34, in the presence or absence of ectopic expression of the wild-type or the *34C68S* mutated version of gene *34* in donor strains (A) or recipient strains (B). The following crosses were performed. For panel A (from left to right), CG159 × PKS7 without IPTG, CG159 × PKS7 with IPTG, CG164 × PS110, PKS11 × PS110, and CG203 × PKS7 without IPTG, and CG203 × PKS7 with IPTG; for panel B (from left to right), PKS11 × CG157 without IPTG, PKS11 × CG157 with IPTG, CG164 × CG157 without IPTG, and CG164 × CG157 with IPTG. Each experiment was repeated at least three times. ANOVAs show that the differences in conjugation efficiencies were statistically significant with *P* values of *P* < 0.0001 for matings of pLS20catΔ34 in the absence or presence of ectopically induced expression of gene *34* in the donor cells, but not in the recipient cells (*P* > 0.05). Although the differences were not statistically significant according to the ANOVA (*P* = 0.0513), a trend was observed that the conjugation levels obtained for the donor strain CG159 grown in the presence of IPTG were about 2-fold higher than those obtained for PKS11, the wild-type strain that harbored pLS20cat. See the legend to Fig. 1 for an explanation of the box plot graph symbols. Strains used: PS110, Spec^r^; PKS7, Em^r^; CG159, *amyE*::P*_hyspank_-34*, pLS20catΔ34; CG164, pLS20catΔ34; PKS11, pLS20cat; CG157, *amyE*::P*_hyspank_-34*; and CG203, *amyE*::P*_hyspank_-34C68S*, pLS20catΔ34.

To test whether ectopic expression of gene *34* could complement the deletion of gene *34* in pLS20cat, we constructed B. subtilis strain CG157 (*amyE*::P*_hyspank_-34*) that allowed conditional expression of pLS20cat gene *34* from the chromosome. Next, pLS20catΔ34 was introduced into CG157 to generate donor strain CG159 (*amyE*::P*_hyspank_-34*, pLS20catΔ34). Low and high conjugation levels were obtained in liquid conjugation experiments in the absence or presence of a 1 mM concentration of the inducer IPTG (isopropyl-β-d-1-thiogalactopyranoside), respectively (Fig. 2A), demonstrating that ectopic expression of gene *34* in donor cells restored efficient conjugation of plasmid pLS20catΔ34. Possible effects of ectopic expression of gene *34* in the recipient cells were also tested, by mating PKS11 (pLS20cat) or CG164 (pLS20catΔ34) with CG157 (*amyE*::P*_hyspank_-34*) recipient cells growing in the absence or presence of a 1 mM concentration of the inducer IPTG. Ectopic expression of gene *34* in recipient cells did not result in increased conjugation efficiency of either pLS20cat or pLS20catΔ34.

### *In silico* analyses of the deduced protein p34 sequence suggests that it is an adhesin.

**FIG 3:**
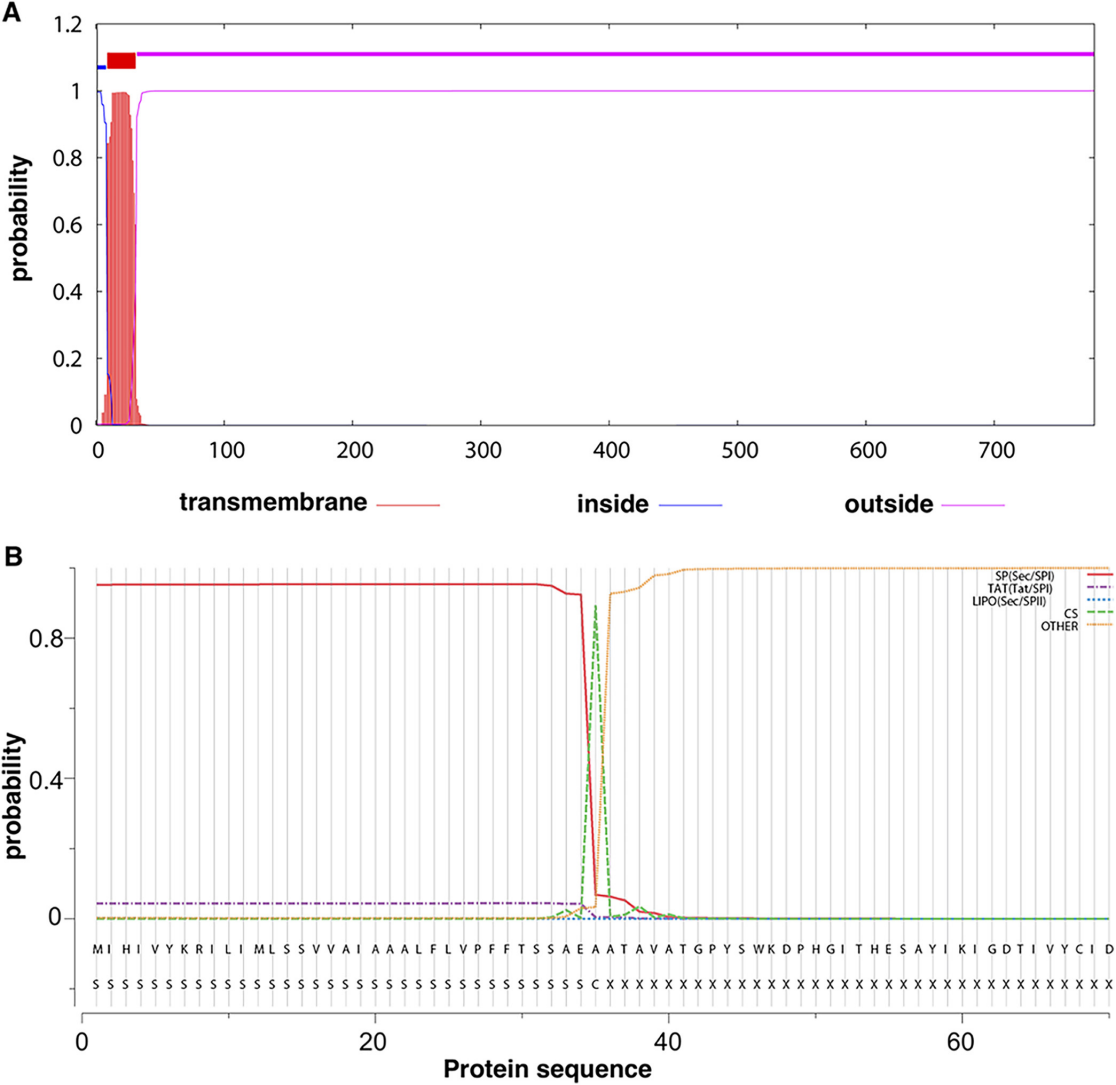
Localization and topology prediction of protein p34. (A) Prediction of transmembrane helices (red) and membrane topology of the pLS20cat-encoded protein p34 (of 778 residues) by the TMHMM 2.0 server (http://www.cbs.dtu.dk/services/TMHMM/) that uses a hidden Markov model (22, 47). (B) Protein p34 signal peptide prediction by the SignalP-5.0 server (http://www.cbs.dtu.dk/services/SignalP/) (23). The horizontal and vertical axes represent the p34 primary sequence and cleavage probability, respectively. The position of the predicted cleavage site (CS) is indicated with a dashed green line.

*In silico* approaches were used to gain insights into features of protein p34 that might explain its importance in conjugation in liquid medium. The deduced sequence of protein p34 was subjected to the TMHMM 2.0 server to predict transmembrane helices and to the signal peptide-screening server SignalP v 5.0 (22, 23). According to these analyses, protein p34 apparently contains a single N-terminal transmembrane-spanning helix (Fig. 3A, residues 9 to 31), which with 95% likelihood represented a signal peptide that would be cleaved between positions 35 and 36 (AEA-AT) by signal peptidase 1 (Fig. 3B). This shows that p34 has features of a protein that will be exported via the Sec-dependent secretion pathway.

Next, we performed a psi-blastp search of the NCBI nr database, using the deduced protein p34 sequence as a query (see Materials and Methods for details). After 16 rounds, this search resulted in the identification of 451 nonredundant hits showing significant similarity with the pLS20cat protein p34 (see Table S1 in the supplemental material).

10.1128/mBio.00104-21.3 TABLE S1 Homologues of pLS20cat protein p34. Download Table S1, XLSX file, 0.02 MB. Copyright © 2021 Gago-Córdoba et al. 2021 Gago-Córdoba et al. https://creativecommons.org/licenses/by/4.0/ This content is distributed under the terms of the Creative Commons Attribution 4.0 International license.

Interestingly, 99.6% of the identified hits corresponded to proteins encoded by bacteria belonging to the phylum *Firmicutes*. A phylogenetic tree was calculated from these 451 hits, which revealed that the identified proteins could be divided into two clades (Fig. S1). Ninety-eight of the 194 hits of the first clade correspond to proteins encoded by bacteria belonging to the Bacillus cereus group, and most of these were annotated as “fusion protein” (including the description “pXO2-28-29-30”). Protein p34 encoded by pLS20cat was assigned to clade 2, which included 257 proteins. Most of the clade 2 hits were annotated as hypothetical proteins. However, several were designated a (putative) function related to conjugation or adhesion, such as conjugal transfer protein (15 hits), isopeptide-forming-domain-containing fimbrial protein (12 hits), MucBP domain (19 hits), and thioester bond-forming surface protein (6 hits). These results suggest that many *Firmicutes* bacteria contain genes for proteins similar to that encoded by pLS20cat gene *34*.

10.1128/mBio.00104-21.2 FIG S1 Phylogenetic tree of 451 homologues of pLS20cat-encoded protein p34. Using the protein pLS20cat-encoded p34 sequence as query in a psi-blastp search, 451 nonredundant hits were obtained after 16 iterations. The three adhesins containing a class II thioester domain for which the structure has been determined were added to this list. Next, these sequences were aligned using blosum62 score as distance with Jalview, and finally the phylogenetic tree was built and plotted with Dendroscope (see also Materials and Methods). Protein p34 of pLS20cat is shown in red. The three adhesins containing a class II thioester domain and for which the structure has been determined are shown in blue. Download FIG S1, PDF file, 0.5 MB. Copyright © 2021 Gago-Córdoba et al. 2021 Gago-Córdoba et al. https://creativecommons.org/licenses/by/4.0/ This content is distributed under the terms of the Creative Commons Attribution 4.0 International license.

We next performed a blastp search against proteins encoded by plasmids present in the PLSDB database using stringent conditions (E value <1E−75, coverage >75%), to see how many other plasmids encoded a protein similar to p34. This search resulted in the identification of 85 homologous proteins of which 22 had >75% coverage, and 11 of these were encoded by unique plasmids (see Table 4 and Fig. S1). These 11 homologous genes were all present within putative conjugation operons (not shown), strongly indicating that these plasmids are conjugative and that the identified genes play similar roles in conjugation as gene *34* for pLS20cat.

In addition, we ran the protein p34 sequence against the HHpred server (24), applying standard parameters and selecting the PDB_mmCIF70 database. This revealed that the p34 region spanning residues 35 to 567 shared significant similarity with regions of 101 proteins, all encoded by G^+^ bacteria. Interestingly, all of them were annotated with one or more of the following keywords: surface protein, adhesin, collagen binding protein, LPXTG-anchored surface protein, thioester domain, pilin subunit, Ig-like fold, CnaA/CnaB folded domains, intramolecular amide bond, fimbrial, subunit, and/or integrin (Table S2). The hit with the highest level of homology and over the longest p34 region (residues 42 to 567, 99.8% probability, E value 1.1e−16) corresponded to the Bacillus anthracis-encoded collagen adhesin protein named BaTIE (PDB 6FWV). This adhesin is a covalently cell wall-anchored protein with an N-terminal signal peptide, which is followed sequentially by a thioester domain (TED), three CnaB domains (see below), a C-terminal LPXTG motif, and a transmembrane-spanning domain. The region of BaTIE predicted to share structural similarity with pLS20cat p34 corresponds to the TED and the three CnaB domains. These HHpred results, combined with the identification of a putative signal peptide and its importance in efficient conjugation in liquid medium, indicate that pLS20cat p34 is a cell wall-associated adhesin with a thioester and CnaA/CnaB domains. A large number of surface proteins from G^+^ bacteria containing a (putative) TED near their N terminus have now been identified. Often, these proteins are composed of multiple domains including TEDs, CnaA/B domains forming isopeptide bonds, and ester domains. Therefore, this family of proteins has been named TIE (thioester, isopeptide, ester) proteins (25). Based on this, we tentatively name pLS20cat gene *34* TIE_pLS20_.

10.1128/mBio.00104-21.4 TABLE S2 HHpred results obtained using full-length pLS20cat p34 protein as query. Download Table S2, DOCX file, 0.02 MB. Copyright © 2021 Gago-Córdoba et al. 2021 Gago-Córdoba et al. https://creativecommons.org/licenses/by/4.0/ This content is distributed under the terms of the Creative Commons Attribution 4.0 International license.

### Evidence that TIE_pLS20_ (p34) contains a thioester domain followed by putative Cna domains.

TEDs form a covalent thioester bond between a Cys and a Gln residue. The thioester-forming Cys residue is normally positioned within a four-residue conserved motif, [YFL]Cϕπ (ϕ and π corresponding to hydrophobic and hydrophilic residues, respectively) (25). The predicted TED of TIE_pLS20_ contains only one Cys residue (Cys68) that is embedded in the [YFL]Cϕπ motif “YCID” located within the predicted TED of TIE_pLS20_. To study if Cys68 was important for TIE_pLS20_ function, the inducible, wild-type copy of *tie_pLS20_* placed in the chromosome, which was used in the complementation experiment above, was replaced by a C68S mutant copy. The C68S mutation would prevent the formation of the Cys-Gln thioester bond by replacing the presumed reactive SH side chain of Cys with the hydroxyl group of Ser. The resulting strain, CG203 (*amyE*::P*_hyspank_-tie_pLS20_C68S*, pLS20catΔ34), was then used as donor in conjugation experiments, in parallel with the control donor strain CG159 containing a wild-type copy of *tie_pLS20_* (*amyE*::P*_hyspank_-tie_pLS20_*, pLS20catΔ34). Efficient conjugation of pLS20catΔ34 was obtained upon ectopic expression of the wild-type but not the mutant version of *tie_pLS20_* (Fig. 2A). These results demonstrate that residue Cys68 is essential for proper functioning of TIE_pLS20_, probably by forming a thioester bond with a Gln residue, and hence that TIE_pLS20_ would possess a TED.

**FIG 4:**
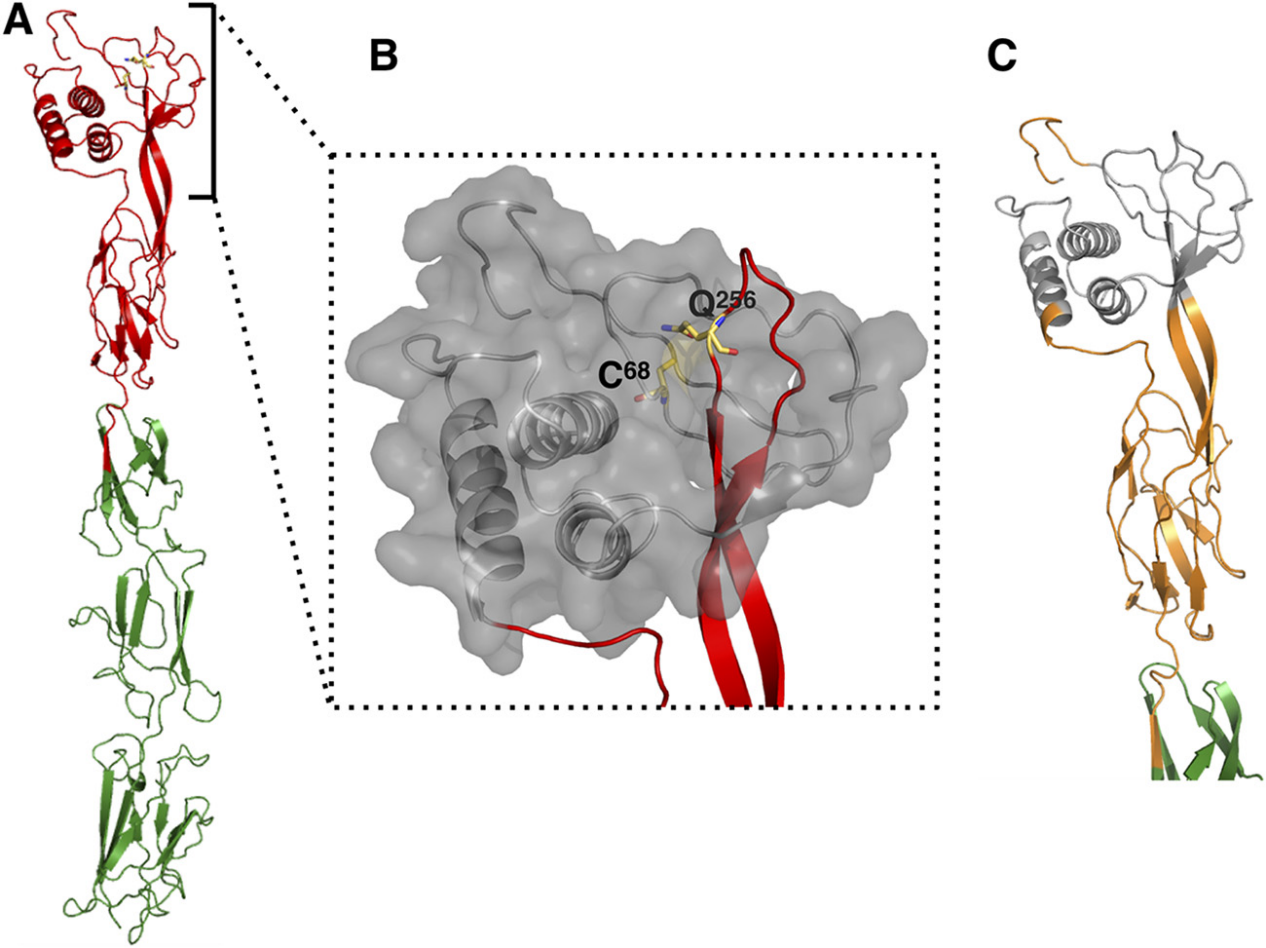
Computer-modeled structure of TIE_pLS20_ and its classification as a class II type TED. (A) Predicted structure built with the MODELLER program of TIE_pLS20_ (residues Tyr44 to Ala567) using as the template-query alignment BaTIE residues 12 to 516 (PDB ID 6FWV); the thioester domain (TED) is shown in red, the thioester-forming bonds Cys68 and Gln256 are shown as yellow sticks, and the Cna domains are shown in green. (B) Slipknot topology representation with its characteristic loop shown in red and the rest of the TED represented in gray with a transparent surface. (C) Structural elements that define TIE_pLS20_ as class II in the TED classification are shown in orange (based on the class II TED structural elements of BaTIE, PDB ID 6FWV) (26).

To determine which Gln residues would form the thioester bond with Cys68, we built a structural model of the presumed TED of TIE_pLS20_ based on the best hit obtained in the above-mentioned HHpred search: the Bacillus anthracis BaTIE protein which contains a class II type TED (26). The model was built with the program MODELLER (27) using as the template-query alignment the one provided by the HHpred search, comprising residues 44 to 567 of the primary TIE_pLS20_ sequence and BaTIE residues 12 to 516 of the 6FWV structure. The BaTIE structure used for building the model includes the TED (6FWV residues 1 to 258) followed by three CnaB domains (domains I, II, and III; 6FWV residues 259 to 343, 344 to 436, and 437 to 526, respectively). The model presented in Fig. 4 shows that Cys68 of TIE_pLS20_ would be located at the end of the slipknot, and the Gln at bond-distance would be Gln256, indicating that Gln256 could form a thioester bond with Cys68. If Gln256 is functionally important, it was expected that this residue would be conserved. Inspection of the alignment of the 451 hits identified by the psi-blastp search revealed that p34 residue Gln256 is indeed highly conserved.

Most TEDs are located near the N terminus of the protein following a secretion signal. Generally, TED-containing adhesins have a large size and are composed of a variety of different domains and/or repeated sequences like isopeptide and ester domains, and fibronectin-binding or proline-rich repeats (25). In BaTIE, the thioester adhesion domain is followed by three structural domains adapting a characteristic sandwich-like structure that is similar to the so-called immunoglobulin domains (Ig domains or Ig-fold). Ig-folds are formed by two facing β-sheets, each being composed of antiparallel β-strands (Fig. 4A) (28). Many adhesins of G^+^ bacteria contain modified Ig-like domains, whose principal “stalk function” is to project the thioester adhesion domain away from the cell surface. The best-studied adhesins containing such folds are those similar to the collagen-binding Cna protein of Staphylococcus aureus (the Cna family) (29, 30), and therefore domains of adhesins containing such folds are also referred to as Cna domains. Interestingly, a characteristic feature of Ig-like folds, including Cna domains, is that, with the help of an adjacent catalytic Glu or Asp residue, they form intramolecular isopeptide bonds between a Lys and either an Asn or an Asp residue. These bonds are located at strategic positions within the protein and provide mechanical strength to the adhesin in order to resist shear forces, thereby ensuring firm adherence of the bacterial cell to its substrate. The isopeptide bonds are always in the hydrophobic interior of the domain, but depending on the strands that are joined, they are named CnaA or CnaB domains.

In BaTIE, the thioester adhesion domain is followed by three CnaB domains in which the isopeptide bonds are formed between Lys297 and Asn373 (domain I), Lys384 and Asp464 (domain II), and Lys475 and Asn555 (domain III). Resides Glu343, Glu443, and Glu524 are the putative catalytic residues for isopeptide bond formation in domains I, II, and III, respectively. According to HHpred, the TIE_pLS20_ region shared structural similarity with a continuous region of BaTIE encompassing the TED and the three subsequent CnaB domains. In the modeled structure of TIE_pLS20_, the region following the thioester domain indeed has three Ig-like folds that are similar to the three CnaB domains in BaTIE. The corresponding domains in TIE_pLS20_ would consist of residues 286 to 349 (domain I), 350 to 438 (domain II), and 439 to 567 (domain III). However, it is presently hard to designate with high confidence the residues that form the isopeptide bonds.

**FIG 5:**
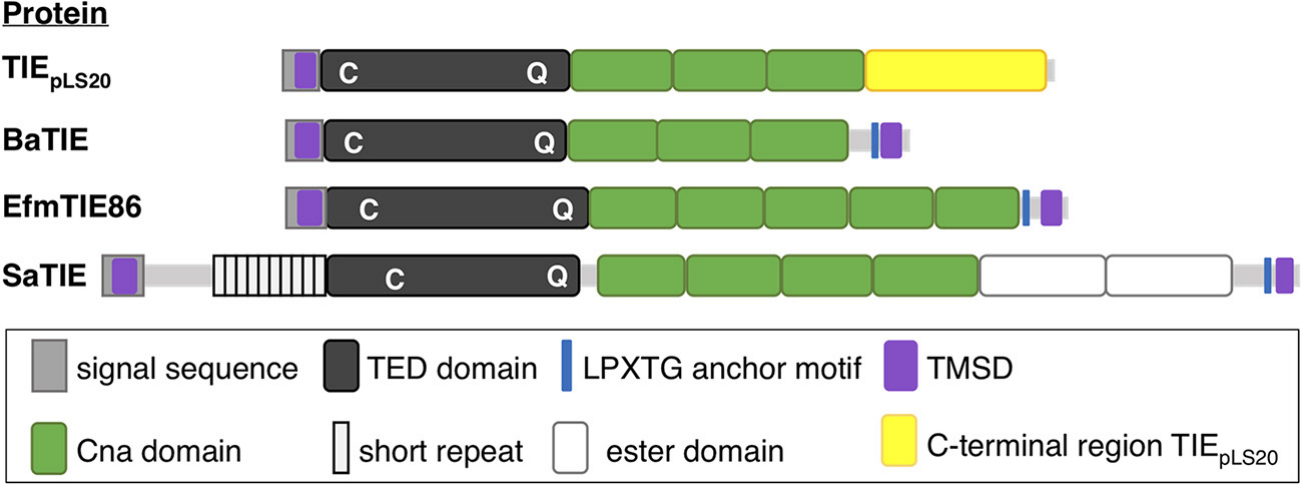
Structural organization of TIE_pLS20_ and TIE proteins encoded by the pathogens B. anthracis, S. aureus, and E. faecium, all containing a class II TED in their N-terminal region. The different domains are indicated with color-coded rectangles, and the proteins are aligned according to their thioester domains. Figures are drawn at scale. TIE_pLS20_, BaTIE, EfmTIE86, and SaTIE are encoded by B. subtilis plasmid pLS20, B. anthracis, E. faecium VRE, and S. aureus VRS11b, respectively. The figure is adapted from Fig. 1A of the work of Miller et al. (26). TMSD, transmembrane-spanning domain.

In summary, TIE_pLS20_ shows a structural organization that is typically found in large multidomain TIE-type adhesins encoded by G^+^ bacteria. A schematic view of the structural organization of TIE_pLS20_ and the class II TED-containing TIE proteins BaTIE, SaTI, and EfmTIE86 is presented in Fig. 5.

### TIE_pLS20_ is not coupled to the cell wall by a sortase.

The structural similarity between TIE_pLS20_ and the cell wall-attached adhesins, such as BaTIE, raised the possibility that TIE_pLS20_ is also covalently attached to the cell wall by a sortase. Surprisingly, though, TIE_pLS20_ does not contain the typical cell wall sorting signal composed of the LPXTG motif at which the sortases act and a C-terminal transmembrane-spanning domain. To investigate this further, we tested whether sortases were required for the function of TIE_pLS20_. *In silico* analysis showed that pLS20 did not contain a sortase gene. The bacterial genomes of most bacilli contain two genes, *yhcS* and *ywpE*, encoding probably functional but nonessential sortases (31, 32). Remarkably, the genome of B. subtilis strain 168 used in our studies contains a deletion affecting the first 81 to 82 codons of *ywpE* and its entire upstream gene. If a sortase is responsible for cell wall attachment of TIE_pLS20_, the *yhcS* gene and/or the truncated *ywpE* gene would be expected to play a role in pLS20 conjugation. We therefore introduced pLS20cat into *yhcS* and *ywpE* single-mutant strains, and an *yhcS*/*ywpE* double mutant strain, and used these as donors in conjugation experiments. As shown in Fig. 1C, high levels of conjugation, similar to those observed for the wild-type donor strain PKS11, were observed. These results show that neither the host-encoded sortase YhcS nor the truncated YwpE proteins are required for linking TIE_pLS20_ to the cell wall.

## DISCUSSION

Mating pair formation is a crucial initial step in the conjugation process and can thus be a target for combating conjugation-mediated spread of antibiotic resistance and virulence genes. So far, little is known about mating pair formation in G^+^ bacteria. The only mating pair system in G^+^ bacteria that has been studied in considerable depth is encoded by the conjugative enterococcal plasmid pCF10 (14). The pCF10-encoded PrgB surface protein is involved in forming mating pair aggregates that are important for efficient conjugation in liquid cultures but not on solid surfaces. It is also a virulence factor promoting attachment and biofilm development of E. faecalis cells on biotic and abiotic surfaces. The PrgB protein is exported via an N-terminal secretion signal and is anchored to the cell wall by its C-proximally located LPTXG motif. One adhesin and two Arg-Gly-Asp (RGD) domains are located in between the signal peptide and the LPXTG motif. The RGD-containing domains are implicated in binding host cell integrins. Cell aggregation and efficient conjugation require, besides PrgB, also extracellular DNA (eDNA) (33). The structure of the PrgB adhesin domain that is responsible for eDNA binding and compaction shares similarity to the lectin-like fold in general and, particularly, to the multimodal *Streptococcus* sp. AglI/II, SspB, and SpaP adhesins (15, 34). It has been proposed that PrgB-mediated compaction of eDNA may serve an analogous function as the retractile conjugative F-pili. Since the G^+^ cell wall component lipoteichoic acid (LTA) competes with eDNA for binding with the adhesion domain, it is envisioned that PrgB first favors cell-cell contact through eDNA compaction and then stabilizes these contacts through LTA binding. Finally, proper functioning of PrgB probably requires also the surface protein PrgA in a yet-unknown manner. Our results show that, in the case of pLS20, mating pair formation is also facilitated by an adhesin encoded by gene *34* of pLS20, which we have named *tie_pLS20_*. Like PrgB, TIE_pLS20_ is important for efficient conjugation, particularly in liquid medium. However, TIE_pLS20_ is structurally very different from PrgB. TIE_pLS20_ has a typical G^+^ adhesin domain architecture with an N-terminal secretion signal, followed by a class II type thioester domain and three structural domains whose functions are probably to direct the adhesin domain away from the donor surface and to provide strength and stability to the large elongated adhesin molecule.

To exert their function, after export from the cytoplasm, surface proteins must be retained on the cell wall. In G^+^ bacteria, there are three known mechanisms that link surface proteins to the cell wall (for review, see references [Bibr B35][Bibr B36][Bibr B37]). First, lipoproteins contain a lipid moiety linked to their N-terminal Cys residue. TIE_pLS20_ does not contain a lipobox, making it highly unlikely that it will become attached to the membrane by N-terminal lipidation. Another reason making this scenario unlikely is that this would position the TED adhesion domain either (partially) in the cell wall or at the cell surface with the Cna domains pointing away from the cell surface. A second way of linking surface proteins to the cell wall is through modules that mediate multiple noncovalent hydrophobic and/or charge-based interactions with cell wall components. Examples of such modules are the GW module, LysM motif, or surface-layer homology domain. TIE_pLS20_ does not contain a module or region showing similarity with any such domains. Finally, a third way by which surface proteins can become attached to the cell wall is through sortase-mediated covalent attachment to the peptidoglycan. These surface proteins contain, besides the N-terminal secretion signal, a C-terminal sorting signal consisting of a conserved LPXTG motif followed by a C-terminal transmembrane domain and a positively charged tail. Sortases act on the LPXTG motif and either link proteins together to form pili or attach large multidomain proteins like TIE proteins to cross-bridge peptides of the cell wall, which are then incorporated as precursors into the cell wall by penicillin-binding proteins. Most surface proteins, including adhesins belonging to the family of “microbial surface components recognizing adhesive matrix molecules” (MSCRAMM) or pilin structures, are covalently anchored to the cell wall in a sortase-dependent way (30, 38, 39). Sortases are also known to attach the three adhesins BaTIE, SaTIE, and EfmTIE86 containing the class II type TED on the cell surface. However, our results indicate that TIE_pLS20_ does not become anchored to the cell wall in a sortase-dependent manner: pLS20 does not carry a sortase gene, and we showed that the conjugation efficiency was not significantly affected using donor strains lacking one or both of the chromosomally located sortase genes. Moreover, TIE_pLS20_ lacks a typical sorting signal. Therefore, the absence of a lipobox, sorting signal, and any known modules that would allow noncovalent interactions with cell wall components raises the intriguing question of how TIE_pLS20_ attaches to the cell wall. The conserved structural organization of G^+^ adhesins and that of TIE_pLS20_ suggest that TIE_pLS20_ remains attached to the cell wall through its C-terminal region, which will direct the TED adhesion module away from the cell surface. According to the RaptorX protein modeling server (40), part of the approximately 250-residue C-terminal region of TIE_pLS20_ shares similarity with a structural domain of unknown function named “toast-rack” (pfam17115) that is present on a putative adhesin encoded by Clostridium sporogenes ATCC 15579 (PDB 4QRK). Although at present we do not know how TIE_pLS20_ becomes attached to the cell wall, based on all the arguments outlined above, it seems plausible that the C-terminal region of TIE_pLS20_ plays a role in this. Future subcellular localization studies are required to verify this hypothesis. Likewise, it will be interesting to investigate how TIE_pLS20_ is targeted to the B. subtilis cell envelope and how exactly this protein facilitates direct contact between the donor and recipient cells for conjugal DNA transfer.

We have also shown that TIE_pLS20_ contains a class II type TED, which is probably responsible for the adhesive properties of the protein. The constructed model indicated that the Tie_pLS20_ residues Cys68 and Gln256 form a thioester bond, which was supported by the fact that the *tie_pLS20_C68S* mutant is not functional in conjugation. Surface-located adhesins and their crucial roles in attachment of commensal and pathogenic bacteria to host surfaces as a prerequisite for colonization and infection have been known for a long time (41). In many of these cases, adhesion of bacteria to a host is based on noncovalent interactions, involving extensive intermolecular regions. The possibility of covalent interaction of a bacterium with a host through a reactive thioester bond was discovered only relatively recently. In 2010, the Cpa pilus tip adhesin from the G^+^ human pathogen Streptococcus pyogenes was shown to contain a thioester bond required for efficient host cell interaction (42). In 2015, Walden et al. showed that many G^+^ adhesins contain a (putative) TED and hence that covalent attachment to the host is more common than previously assumed (25). They also revealed structures of class I TEDs and demonstrated that the streptococcal adhesin SfbI reacts with one specific fibrinogen lysine residue in a thioester-dependent mechanism, resulting in a very stable intermolecular amide bond (25). Determination of class II TED structures revealed that they contain, compared to class I TEDs, an additional β-sandwich domain that forms a slipknot structure with the conserved TED fold. The structures of the class II TEDs have been reported for three pathogens, B. anthracis and vancomycin-resistant strains of S. aureus and Enterococcus faecium (26). TEDs thus appear to be common adhesin molecules permitting covalent attachment of many pathogens to host cells. However, this is the first time that a TED-containing adhesin has been shown to play a role in conjugation.

Database searches revealed that several other conjugative plasmids of G^+^ bacteria also contain a class II type TED-containing adhesin, suggesting that mating pair formation is also mediated by the TED domain in these cases. In fact, it is probable that mating pair formation in G^+^ bacteria is mediated by adhesins in general. In this respect, it is interesting that antiadhesion therapies have been studied for more than 2 decades as a way to combat bacterial infections that are resistant to antibiotics. These therapies include the use of receptor and adhesin analogues, dietary constituents, and adhesin-based vaccines (43, 44). It is possible that similar strategies can be applied to curtail adhesin-based mating pair formation, thereby impeding conjugation-mediated spread of antibiotic resistance and virulence genes.

## MATERIALS AND METHODS

### Bacterial strains, plasmids, media, and oligonucleotides.

B. subtilis and E. coli strains were grown and selected in lysogeny broth (LB), or on 1.5% LB agar plates. When appropriate, media were supplemented with the following antibiotics: ampicillin (100 μg/ml), spectinomycin (100 μg/ml), chloramphenicol (5 μg/ml), erythromycin (1 and 150 μg/ml for B. subtilis and E. coli, respectively), and kanamycin (10 and 30 μg/ml for B. subtilis and E. coli, respectively). For induction of the P*_spank_* and P*_hyspank_* promoters and the P*_xyl_* promoter, media were supplemented with 1 mM isopropyl-β-d-1-thiogalactopyranoside (IPTG) or 1% xylose, respectively. Antibiotics and chemicals were purchased from Sigma-Aldrich, oligonucleotides were from Isogen (Life Science, The Netherlands), and restriction enzymes and T4 DNA ligase were from New England Biolabs (NEB). All B. subtilis strains used were isogenic with B. subtilis strain 168 (Bacillus Genetic Stock Centre [Table 1]). Plasmids and oligonucleotides are listed in Tables 2 and 3, respectively.

**TABLE 1:** Strains used

Bacterium	Strain	Genotype	Reference and/or source
E. coli	XL1-Blue	*endA1 gyrA96(nalR) thi-1 recA1 relA1 lac glnV44 F*′*[*::Tn*10 proAB*+ *lacIq* Δ*(lacZ)M15] hsdR17(rK*− *mK+)*	56
	JM101	*F*′ *traD36 proA+B+ lacIq* Δ*(lacZ)M15/*Δ*(lac-proAB) glnV thi*	57

B. subtilis	168 (1A700)	*trpC2*	BGSC^*a*^
	PS110	*trpC2*, *amyE*::P*_spank_*-Δ (*spec*)	19
	PKS7	*trpC2*, *thrC*::*Em*	19
	PKS11	*trpC2*, pLS20cat	58
	PKS91	*trpC2*, pLS20spec	21
	CG164	*trpC2*, pLS20catΔ34	This work
	CG157	*trpC2*, *amyE*::P*_hyspank_-34*/*tie_pLS20_* (*spec*)	This work
	CG202	*trpC2*, *amyE*::P*_hyspank_-34*/*tie_pLS20_C68S* (*spec*)	This work
	CG159	*trpC2*, *amyE*::P*_hyspank_-34*/*tie_pLS20_* (*spec*), pLS20catΔ34 (*cat*)	This work
	CG203	*trpC2*, *amyE*::P*_hyspank_-34*/*tie_pLS20_C68S* (*spec*), pLS20catΔ34 (*cat*)	This work
	BKE09200	*trpC2*, *yhcS*::*Em*	BGSC, 59
	BKK09200	*trpC2*, *yhc*::*Km*	BGSC, 59
	BKE36340	*trpC2*, *ywpE*::*Em*	BGSC, 59
	BKK36340	*trpC2*, *ywpE*:*Km*	BGSC, 59
	CG265	*trpC2*, *yhcS*::*Em*, pLS20cat	This work
	CG266	*trpC2*, *yhcS*::*Km*, pLS20cat	This work
	CG267	*trpC2*, *ywpE*::*Em*, pLS20cat	This work
	CG268	*trpC2*, *ywpE*::*Km*, pLS20cat	This work
	CG269	*trpC2*, *ywpE*::*Km*, *yhcS*::*Em*	This work
	CG271	*trpC2*, *ywpE*::*Km*, *yhcS*::*Em*, pLS20cat	This work

a BGSC, Bacillus Genetic Stock Centre.

**TABLE 2:** Plasmids used

Plasmid name	Description	Reference
pLS20cat	Native plasmid pLS20 labeled with Cm resistance gene in unique SalI site	Laboratory stock
pLS20catΔ34	Derivative of pLS20cat containing large internal deletion of gene *34*/*tie_pLS20_*	This work
pMiniMAD2	Plasmid used for markerless deletions	Gift of Daniel Kearns
pCGdelta34	pMiniMAD2 derivative to create in-frame markerless partial deletion (from codon 285 to 605) of pLS20cat gene *34*/*tie_pLS20_*	This work
pDR110	B. subtilis *amyE* integration vector containing IPTG-inducible P*_spank_* promoter	Gift of David Rudner
pDR111	B. subtilis *amyE* integration vector containing IPTG-inducible P*_hyspank_* promoter	Gift of David Rudner
pCG197	pDR111 derivative containing a 729-bp fragment coding for the N-terminal region of gene *34*/*tie_pLS20_* harboring the C68S mutation behind the P*_hyspank_* promoter	This work
pCG205	pDR111 derivative containing the complete pLS20cat gene *34*/*tie_pLS20_* harboring C68S mutation behind the P*_hyspank_* promoter	This work

**TABLE 3:** Oligonucleotides used

Name	Sequence (5′–3′)^*a*^	Purpose
oEST15	tttt**gtcgac**GACAATAAGAAAGGAGGTGATCGAAGTGATTC	Forward oligo^*b*^ to amplify gene *34*/*tie_pLS20_* and to be cloned in pDR110/11, includes SalI site
oEST16	tttt**gcatgc**CCTCCAAACAGTTGAAAAGGTTATTTCTTCAACC	Reverse oligo to amplify gene *34*/*tie_pLS20_* and to be cloned in pDR110/11, includes SphI site
pDR111_U_sec	TGACTTTATCTACAAGGTGTGGC	Oligo used for sequencing, and colony PCR of derivatives of pDR110/11
pDR111_L_sec	TTAAATGCAACCGTTTTTTCGGAAGG	Oligo used for sequencing, check colony PCR of derivatives of pDR110/11
LS20_8	AGTTATGACAGAAGTAACCCCAACA	Internal oligo of gene *34*/*tie_pLS20_* used for sequencing, and colony PCR
LS20_4Back	CTTTCCATGAATACGGACCTGTGGC	Internal oligo of gene *34*/*tie_pLS20_* used for sequencing, and colony PCR
oCG49	CCCCATCGGGATTTGTTTCTTTG	Oligo used for sequencing and colony PCR of derivatives of pMiniMAD2
oCG50	aaaa**gtcgac**GGTGACGGTAATGAAATTGGA	Forward oligo to amplify *34*/*tie_pLS20_* “UP” region in combination with oligo oCG51. It also contains an NheI restriction site extension used for cloning of the PCR fragment in pMiniMAD2
oCG51	CTCTTTTACTTTCTTTAAGACCGTGCCTTC*GAATTTGATATCATCATCGTC*	Reverse oligo to amplify *34*/*tie_pLS20_* “UP” region in combination with oligo oCG50. Contains a 5′ extension used in subsequent overlapping PCR to fuse the *34*/*tie_pLS20_* “UP” region with the “DOWN” region
oCG52	*GACGATGATGATATCAAATTC*GATTTTAAAGTACCTGAAG	Forward oligo to amplify *34*/*tie_pLS20_* “DOWN” region in combination with oligo oCG53. Contains a 5′ extension used in subsequent overlapping PCR to fuse the *34*/*tie_pLS20_* “DOWN” region with the “UP” region
oCG53	tttt**ggatcc**GAGTCTCGTTATTTTTGTGATGGC	Reverse oligo to amplify *34*/*tie_pLS20_* “DOWN” region in combination with oligo oCG52. It also contains a BamHI restriction site extension used for cloning of the PCR fragment in pMiniMAD2
oCG54	GTCTACTTAGCTCTTTTTTCACAATAC	Oligo used for sequence analysis and colony PCR of derivatives of pMiniMAD2
oCG124	GCTTATCTGGATCAAT*ACT*GTAAACAATGGTGTCTCCTATTTTGATGTAAGCTGATTCATGAGTAATGC	Reverse oligo to generate “UP” fragment that contains the mutation in gene *34*/*tie_pLS20_* to change codon Cys68 into Ser68 (indicated in italic). Used in combination with oEST15
oCG125	CCATTGTTTAC*AGT*ATTGATCCAGATAAGCCTGCACCTTATGGCGGTCATTCGTATAAAACCCCGAAGCGT	Forward oligo to generate “DOWN” fragment that contains the mutation in gene *34*/*tie_pLS20_* where codon Cys68 is substituted by Ser68 (indicated in italic). Used in combination with oCG126
oCG126	aaaa**gcatgc**ttaCGTAGTCAGATCCATAATCTAAAGGTGC	Reverse oligo to amplify the “DOWN” region of gene *34*/*tie_pLS20_*. It contains a 5′ extension with SphI restriction site; used in combination with oCG125
oCG141	GGCAGCTCTGTTTCCGGTGATCAGCAAAAATCAGAAACCATTAAGC	Forward oligo to amplify gene *34*/*tie_pLS20_* starting from codon 167. Used in combination with oEST16

a Capital letters, pLS20 sequences; bold, restriction enzyme sites; underlining, stop codons (also underlined); italic, overlapping sequences.

b oligo, oligonucleotide.

### Transformation.

E. coli cells were transformed by standard procedures (45). Generation of competent B. subtilis cells and natural transformations were done as previously described (46).

### Conjugation assays.

Conjugation in liquid medium was carried out as described previously (19). Conjugation in solid medium was performed similarly but with the following changes. Samples of late exponentially growing donor and recipient cultures (optical density at 600 nm [OD_600_] between 0.8 and 1.0) were mixed at 1:1 stoichiometry and spread on nonselective plates. Part of the mixture was plated on selective plates, to verify the 1:1 stoichiometry of donor and recipient cells. Cells grown overnight on a nonselective plate were harvested, and dilutions were spread on selective plates to select for donors and transconjugants.

### Construction of plasmids and strains.

Details on the construction of plasmids and strains are given in Text S1 in the supplemental material. Table 4 shows conjugative plasmids of G^+^ bacteria encoding a pLS20cat p34 homologue.

**TABLE 4:** Conjugative plasmids of G^+^ bacteria encoding a pLS20cat p34 homologue

Bacterial strain	Plasmid name	Plasmid identifier	Protein identifier	% identity	Length alignment (residues)	MM^*a*^	Gaps	q start^*b*^	q end^*c*^	S start^*d*^	S end^*e*^	E value	Bit score^*f*^
B. subtilis natto	pLS20	NC_015148.1	YP_004243498.1	99.9	753	1	0	26	778	1	753	0	1139
B. subtilis KH2	NG^*g*^	NZ_CP018185.1	WP_033881018.1	99.9	777	1	0	2	778	1	777	0	1179
B. subtilis SRCM100333	pBS333	NZ_CP021893.1	WP_088272629.1	100	767	0	0	12	778	1	767	0	1160
Bacillus velezensis DSYZ	pDSYZ	NZ_CP030151.1	WP_082188812.1	77.7	767	171	0	12	778	1	767	0	1146
Listeria grayi	pLGUG1	NC_014496.1	YP_003877896.1	51.4	775	370	3	4	778	2	769	0	1145
Listeria monocytogenes R479a	pLMR479a	NZ_HG813248.1	WP_025370673.1	51.0	774	372	3	4	777	2	768	0	1137
Listeria monocytogenes FDA00006907	pCFSAN021445	NZ_CP022021.1	WP_003725232.1	51.9	774	363	6	5	778	3	767	0	1102
Listeria monocytogenes HPB5415	pLM5578	NZ_CP019166.1	WP_012952147.1	51.7	770	362	6	11	777	7	769	0	1091
Bacillus velezensis GH1-13	NG*^g^*	NZ_CP019039.1	WP_077721679.1	58.7	762	308	6	20	778	3	760	0	1063
Bacillus safensis U14-5	NG^*g*^	NZ_CP015608.1	WP_075623813.1	52.3	767	359	6	15	778	8	770	0	1035
Terribacillus goriensis MP602	pT1	NZ_CP008877.1	WP_041592320.1	35.1	764	473	12	30	777	111	867	0	885

a MM, mismatches.

b Start alignment query sequence.

c End alignment query sequence.

d Start sequence hit.

e End sequence.

f Bit score.

g Not given. Note that this search identified the p34 sequence of pLS20cat as deposited in the NCBI database, and hence, it is 25 residues shorter (see first paragraph of Results).

10.1128/mBio.00104-21.1 TEXT S1 Detailed description of strains and plasmids. Download Text S1, DOCX file, 0.02 MB. Copyright © 2021 Gago-Córdoba et al. 2021 Gago-Córdoba et al. https://creativecommons.org/licenses/by/4.0/ This content is distributed under the terms of the Creative Commons Attribution 4.0 International license.

### *In silico* analyses. (i) Identification of membrane, secreted, and surface proteins.

Deduced pLS20cat protein sequences were screened for the presence of transmembrane-spanning domains and their transmembrane topology using the TMHMM server version 2.0 of the Centre for Biological Sequence Analyses at the Danish Technical University (DTU; www.cbs.dtu.dk/services/TMHMM) (22, 47). The presence of potential signal peptidase 1 cleavage sites was predicted using the SignalP-5.0 server (http://www.cbs.dtu.dk/services/SignalP/) with default settings for proteins of G^+^ bacteria.

### (ii) Identification of genes encoding proteins with significant similarity to pLS20cat protein p34.

The primary sequence of the pLS20cat-encoded protein p34 was used as a query sequence to execute psi-blastp searches (https://blast.ncbi.nlm.nih.gov/Blast.cgi?PAGE=Proteins) against the NCBI nr protein database (version 2.10.0+, February 2020) (48–50). Iterative rounds of psiBlast searches were done until no new sequences with an E value of <1E−75 and a coverage of >75% of the entire length of the query sequence were incorporated into the psiBlast profile, which occurred after 16 rounds. All sequences with an E value of <1E−20 and a coverage of >75% were retrieved for subsequent analysis.

### (iii) Generation of a phylogenetic tree.

First, protein sequences of all 451 proteins with similarity to p34 were aligned using the program “decipher” with default settings (51). Next, an average linked tree was built using blosum62 similarity score as distance with the program Jalview (52, 53). The resulting phylogenetic tree was plotted with Dendroscope (54).

### (iv) Identifying protein p34 homologues encoded by plasmids.

We generated a database containing all protein sequences encoded by plasmids present in the PLSDB, a plasmid database (55). Next, the p34 protein sequence was used as query to perform a blastp search against this protein database.

### (v) Structure prediction and modeling.

Sequences of interest were submitted for online predictions to the following servers: HHpred (https://toolkit.tuebingen.mpg.de/tools/hhpred) (24), MODELLER (https://salilab.org/modeller/) (27), and RaptorX (http://raptorx.uchicago.edu/) (40). Analyses were performed using standard parameters.
