# Supplementary material for: A Conserved Class II Type Thioester Domain-Containing Adhesin Is Required for Efficient Conjugation in Bacillus subtilis
Source: mBio. 2021 Mar 16;12(2):e00104-21. doi: 10.1128/mBio.00104-21 (PMC8092201; doi:10.1128/mBio.00104-21)
Supplement: TABLE S2 [file mBio.00104-21-st002.docx]

| Supplemental TABLE S2. HHPred results obtained using full-length pLS20cat p34 protein as query. | | | | | | | | | |
| --- | --- | --- | --- | --- | --- | --- | --- | --- | --- |
| **hits** | **organism** | **Protein function and Motifs** | **PDB / Uniprot** | **Prob^§^**  **(%)** | **E-value** | **Target length** | **FL Protein length*** | **Query start** | **Query ends** |
| 1 | *Bacillus anthracis* | Collagen Adhesion protein BaTIE-TED. (LPTXTG Surface anchored protein,Isopeptide bond and TED domain) | 6FWV_A  A0A0F7RA58 | 99.86 | 3.6e-17 | 526 | 627 | 42  (44) | 509  (542) |
| 2 | *Staphylococcus aureus* | Collagen Adhesion protein SaTIE-TED. (LPTXTG Surface anchored protein, and TED domain) | 6FX6_A  A0A3F2YM24 | 99.72 | 3.3e-16 | 253 | 943 | 53  (1) | 294  (22) |
| 3 | *Enterococcus faecium* | Collagen Adhesion protein TIE86.(LPTXTG Surface anchored protein,Isopeptide bond and TED domain) | 6FWY_D  A0A1A7T0E1 | 99.47 | 4e-12 | 280 | 796 | 62  (80) | 280  (314) |
| 4 | *Lactobacillus rhamnosus GG* | Cell surface protein SpaA. (Isopeptide containing basal pilin from SpaFED pilus) | 6JCH_A  A0A5H1ZR38 | 97.68 | 0.00034 | 400 | 400 | 280  (9) | 450  (351) |
| 5 | *Bacillus cereus* | Collagen adhesion protein. (Intramolecular amide bond containing pilin subunit) | 3KPT_A  Q81D71 | 97.9 | 0.00048 | 355 | 553 | 280  (166) | 428  (511) |
| 6 | *Streptococcus pyogenes* | Fibronectin-binding protein  (TED domain of fibronectin-binding protein SfbI) | 5A0L_  A0A0M3KL43 | 96.64 | 0.0054 | 210 | 210 | 62  (43) | 128  (137) |
| 7 | *Corynebacterium diphtheriae* | Putative fimbrial subunit. (Major pilin of SpaD) | 4HSS_B  Q6NK05 | 97.73 | 0.0062 | 431 | 490 | 269  (40) | 513  (429) |
| 8 | *Clostridium perfringens* | Putative surface anchored protein. (TED domain containing protein) | 5A0D_B  B1R775 | 96.23 | 0.011 | 186 | 1,999 | 62  (132) | 128 (225) |
| 9 | *Streptococcus pyogenes* | Pilus tip adhesin Cpa. (TED domain of pilus tip adhesin Cpa) | 4C0Z_C  S5FV19 | 96.21 | 0.011 | 215 | 680 | 62  (56) | 128  (147) |
| 10 | *Streptococcus pyogenes* | Trypsin-resistant surface T6 protein. (Structural domain of backbone pilin) | 4P0D_A  P18481 | 96.56 | 0.012 | 489 | 537 | 281  (337) | 378  (457) |
| 11 | *Lactobacillus rhamnosus GG* | Cell surface protein SpaA. (Isopeptide of pilin subunit spa) | 5FAA_A  Unknown | 96.86 | 0.018 | 283 | Unknown | 281  (13) | 419  (262) |
| 12 | *Streptococcus equi subsp. equi* | Fibronectin-binding protein. (TED domain containing protein) | 5DCQ_F  Q93ED6 | 95.72 | 0.023 | 271 | 298 | 62  (65) | 128  (161) |
| 13 | *Lactobacillus rhamnosus GG* | Cell surface protein SpaA. (Isopeptide containing basal pilin from SpaFED pilus) | 6JCH_A  A0A5H1ZR38 | 94.82 | 0.062 | 400 | 400 | 270  (186) | 450  (351) |
| 14 | *Streptococcus pneumoniae* | Cell wall surface anchor family. (Structural protein of Major pilin) | 2Y1V_B  A0A0H2UNM7 | 95.9 | 0.11 | 605 | 665 | 280  (341) | 419  (598) |
| 15 | *Bacillus anthracis* | Collagen Adhesion protein BaTIE-TED. (LPTXTG Surface anchored protein, Isopeptide bond and TED domain) | 6FWV_A  A0A0F7RA58 | 97.09 | 0.18 | 526 | 627 | 196  (295) | 424  (555) |
| 16 | *Bacillus cereus* | Collagen adhesion protein. (Intramolecular amide bond containing pilin subunit) | 3KPT_A  Q81D71 | 94.75 | 0.18 | 355 | 553 | 275  (404) | 363  (515) |
| 17 | *Streptococcus agalactiae* | Cell wall surface anchor family protein. (Major Pilin GBS80 of C-terminal fragment) | 3PF2_A  Q8E0S9 | 93.31 | 0.2 | 319 | 554 | 282  (381) | 349  (492) |
| 18 | *Clostridium perfringens* | Probable surface protein CppA. (Pilin protein) | 5XCC_A  Q8XP10 | 93.66 | 0.21 | 476 | 522 | 281  (337) | 349  (430) |
| 19 | *Lactobacillus rhamnosus GG* | Pilin SpaD. (Shaft pilin spaD) | 5YU5_A  Unknown | 94.21 | 0.25 | 465 | Unknown | 282  (331) | 368 (457) |
| 20 | *Corynebacterium diphtheriae* | Putative surface-anchored fimbrial subunit. (Major pilin SpaA isopeptide bonds) | 3HR6_A  Q6NF81 | 93.13 | 0.31 | 436 | 525 | 282  (357) | 349  (467) |
| This table lists the first 20 hits obtained by the HHPred search. *FL, Full length; § prob, probability; | | | | | | | | | |
