# Supplementary material for: A Conserved Class II Type Thioester Domain-Containing Adhesin Is Required for Efficient Conjugation in Bacillus subtilis
Source: mBio. 2021 Mar 16;12(2):e00104-21. doi: 10.1128/mBio.00104-21 (PMC8092201; doi:10.1128/mBio.00104-21)
Supplement: TEXT S1 [file mBio.00104-21-s0001.docx]

**Supplemental Materials and Methods**

*In frame deletion of pLS20cat gene 34.* Marker-less *in-frame* deletions of gene *34* on pLS20cat was constructed using pMiniMAD2 vector (J. E. Patrick, and D. B. Kearns, Mol Microbiol 70:1166-1179, 2008, DOI: 10.1111/j.1365-2958.2008.06469.x). A detailed description of the marker-less in-frame deletion strategy has been given elsewhere(C. Gago-Cordoba, J. Val-Calvo, A. Miguel-Arribas, E. Serrano, P.K. Singh, D. Abia, L. J. Wu, and W. J. J. Meijer, Front Microbiol 10:1502, 2019, doi: 10.3389/fmicb.2019.01502). The following primer sets were used to generate a large internal deletion of pLS20cat gene *34*: [oCG50 and oCG51] and [oCG52 and oCG53] were used to generate the upstream (UP) and downstream (Dn) fragments, respectively. The two fragments were fused in two subsequent PCR reactions. First, 100 ng of each purified PCR fragment were mixed and used as template in the first PCR reaction (50 μl) lacking primers (settings [(4 min 94 ⁰C); 30 rounds (30 sec 94 ⁰C; 30 sec 55 ⁰C; 1.5 min 73 ⁰C)]). Next, 2 μl of this elongation reaction was used as template in a subsequent conventional PCR reaction (100 μl) containing the appropriate outer primers (settings [(4 min 94 ⁰C); 30 rounds (30 sec 94 ⁰C; 30 sec 55 ⁰C; 1.5 min 73 ⁰C)]). The resulting fused PCR product was purified and digested with *HindIII* and *SalI*, then cloned into the pMiniMAD2 vector digested with the same enzymes. The primer set [oCG49-oCG54] was used to check deletion of the desired region in pLS20cat by PCR, using as template total DNA of the erythromycin-sensitive clones. The resulting derivative pLS20catΔ34 lacks gene *34* codons 285 to 605.

*Strains allowing ectopic expression of the wild type pLS20cat gene 34 or the C68S derivative controlled by the IPTG-inducible P_hyspank_ promoter.* The pLS20cat gene *34* was amplified by PCR using primer set [oEST15-oEST16]. The purified PCR fragment was digested with *SalI* and *SphI* and then used to generate a ligation mixture together with the *amyE* integration vector pDR111 digested with the same enzymes. Multiple attempts were made to obtain a derivative of pDR111 containing pLS20cat gene *34* cloned behind the P*_hyspank_* promoter on pDR111 by transforming the ligation mixture into competent *E. coli* cells. However, in all transformants analysed, rearrangements and deletions in gene *34* had occurred and the desired construct was not obtained. Therefore, the ligation mixture was used directly to transform competent *B. subtilis* cells. Spectinomycin-resistant transformants were transferred to LB plates containing starch to select for *amyE*^-^ transformants resulted from double-crossover events that disrupted the *amyE* gene. Integration of the *P_hyspank_-34* construct at *amyE* in the *amyE^-^* and spectinomycin-resistant transformants was confirmed by sequencing the PCR products generated using the primer set [pDR111_U_sec- 34_F2_Dn]. The primers used for sequencing were pDR111_U_sec; LS20_4Back; LS20_8; 34_F2_Dn; 34_F1_Up; and pDR111_L_sec. A representative strain shown to be correct was named CG157 (*amyE:: P_hyspank_ -34*).

The following strategy was used to construct strain CG202 (*amyE:: P_hyspank_-34C68S*). The mutant version of gene *34* in which the cysteine codon (TGT) was altered into the serine codon (AGT) was generated as follows. First, a 741 bp PCR fragment was generated corresponding to the N-terminal region of gene *34* and containing the TGT to AGT mutation at codon 68. For this, two overlapping PCR fragments were generated. The 255 bp “UP” fragment contained the TGT to AGT mutation at its 3´ end, and the 503 bp “DOWN” fragment contained this mutation at its 5´ end. The “UP” and “DOWN” fragments were generated using pLS20cat DNA as template in combination with primer sets [oEST15-oCG124, oEST15 contains a mutation to improve the RBS of gene 34] and [oCG125-oCG126], respectively. Primer oCG126 contains an extension of a TAA stop codon located immediately upstream its 5´ located *SphI* site. Next, these two fragments were fused by PCR using the same strategy as described for the generation of the in frame deletion in gene *34* (see above). The resulting 729 bp PCR fragment was digested with *SalI* and *SphI* and cloned into the pDR111 vector digested with the same enzymes. The resulting plasmid, named pCG197, was propagated without problems in *E. coli*. The region cloned in pCG197 corresponding to the N-terminal region of gene *34* contains a unique *PmlI* site that is located downstream of the introduced mutation. This *PmlI* site was used for a second step to add the remaining part of gene *34*. Thus, primer set [oCG141-oEST16] was used to amplify an 1846 bp fragment corresponding to the 3´ region of gene 34. This fragment was digested with PmlI and SphI and cloned into vector pCG197 digested with the same enzymes. The resulting plasmid named pCG205 was obtained without problems after transforming competent E. coli cells with the corresponding ligation mixture. Correctness of gene 34 and confirmation of its mutation generating the C68S change was confirmed by sequencing. Plasmid pCG205 isolated from E. coli cells was used to transform competent B. subtilis 168 cells. Spectinomycin-resistant transformants were checked for double cross over by the assay to determine loss of function of the amyE gene. The resulting strain was named CG202 (*amyE*:: P_hyspank_-34C68S). Plasmid pLS20catΔ34 was introduced into strains CG202 and CG157 by conjugation using CG164 as donor strain, resulting in strains CG159 (*amyE*:: P_hyspank_-34, pLS20catΔ34), and CG203 (*amyE*:: P_hyspank_-34C68S, pLS20catΔ34).

*Generation of a sortase-deficient B. subtilis strain containing pLS20cat.* The sortase (*yhcS*/*ywpE*) double mutant was obtained by transforming competent cells of BKK36340 (*ywpE::Km*) with DNA of BKE09200 (*yhcS::Em*) and selecting transformants for erythromycin and kanamycin resistance. pLS20cat was then introduced into the sortase double deletion strain by conjugation.
