# Supplementary material for: A Conserved Class II Type Thioester Domain-Containing Adhesin Is Required for Efficient Conjugation in Bacillus subtilis
Source: mBio. 2021 Mar 16;12(2):e00104-21. doi: 10.1128/mBio.00104-21 (PMC8092201; doi:10.1128/mBio.00104-21)

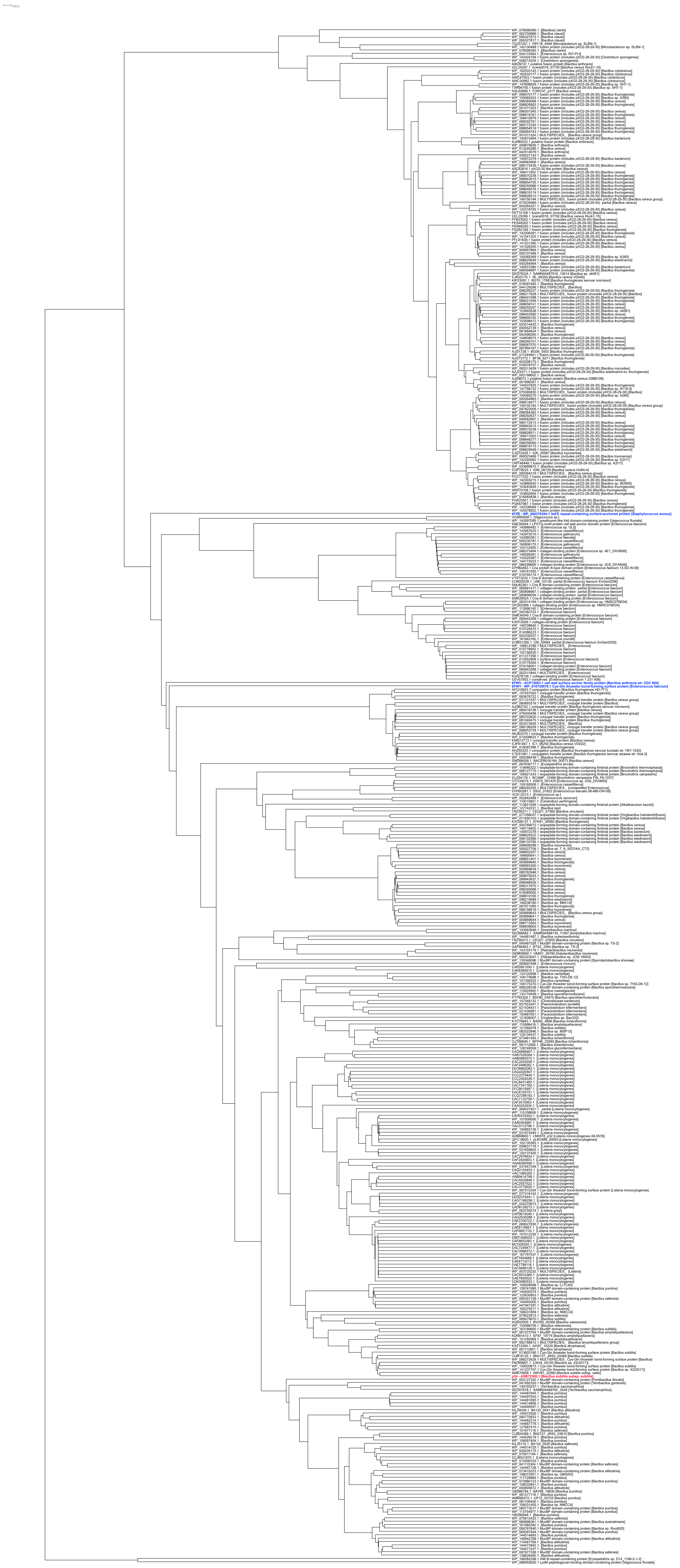

Supplement: FIG S1 [file mBio.00104-21-sf001.pdf]
